# Bed-Embedded Heart and Respiration Rates Detection by Longitudinal Ballistocardiography and Pattern Recognition

**DOI:** 10.3390/s19061451

**Published:** 2019-03-25

**Authors:** Almothana Albukhari, Frederico Lima, Ulrich Mescheder

**Affiliations:** Medical and Mechanical Engineering Faculty, and Institute for Microsystem Technology (iMST), Furtwangen University, 78120 Furtwangen, Germany; lima@hs-furtwangen.de (F.L.); mes@hs-furtwangen.de (U.M.)

**Keywords:** ballistocardiography, vital signs monitoring, contactless monitoring, heart rate, load cell, pattern recognition, unsupervised machine learning, clustering

## Abstract

In this work, a low-cost, off-the-shelf load cell is installed on a typical hospital bed and implemented to measure the longitudinal ballistocardiogram (BCG) in order to evaluate its utility for successful contactless monitoring of heart and respiration rates. The major focus is placed on the beat-to-beat heart rate monitoring task, for which an unsupervised machine learning algorithm is employed, while its performance is compared to an electrocardiogram (ECG) signal that serves as a reference. The algorithm is a modified version of a previously published one, which had successfully detected 49.2% of recorded heartbeats. However, the presented system was tested with seven volunteers and four different lying positions, and obtained an improved overall detection rate of 83.9%.

## 1. Introduction

In 2015 the number of people aged over 60 years reached almost 900 million worldwide, and between 2015 and 2050 that number is forecast to increase by 58% in higher income countries and by higher rates in lower income countries reaching 239% increase in countries at the lower end of the global income spectrum. This demographic shift and the increased life expectancy is associated with increased prevalence of chronic diseases like dementia, from which in 2015 around 46.8 million people had suffered, with a projection of this number almost doubling every 20 years [1].

This epidemic has inspired some new research trends in the medical field, particularly regarding sensory modalities that can be utilized in monitoring the status of dementia patients and their vital signs, because the type of sensor will determine the level of intrusiveness on a patient’s life and daily routines. This is not only true on the hospital bed but also in home care applications, where incorporating monitoring systems could have major benefits for patients as well as caregivers; financially, psychologically, and most importantly in terms of quality of provided healthcare [2]. The development of unobtrusive monitoring systems that cause the least amount of discomfort, while being as ubiquitous in the patients’ environment as possible, is a goal of many recent research efforts, which could accommodate the special conditions of this growing segment of society. Moreover, tracking the cardiopulmonary activity of patients is paramount for determining their well-being and a key indicator for detecting distress situations of patients with dementia.

**Figure 1:**
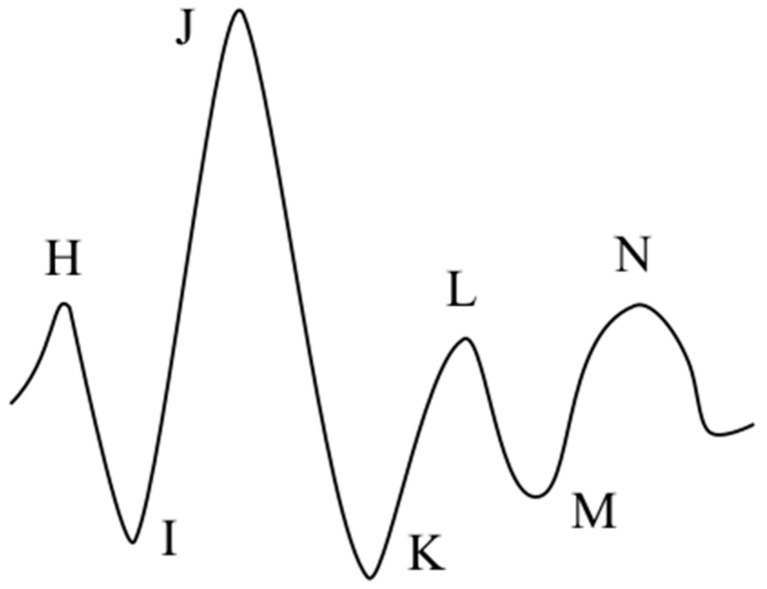
A Diagram of the commonest form of the normal ballistocardiogram with the letters denoting its various waves (tips), redrawn after Starr’s publication [6].

One of the most promising unobtrusive techniques, which has recently regained considerable attraction among researchers, is ballistocardiography, which is the measurement of the body recoil forces resulting from the ejection of blood in the vasculature at each cardiac cycle. BCG was first observed by Gordon in 1877, when he noticed the index of a weighing scale having rhythmic movement synchronously with the pulse of a subject standing vertically on it, he then reproduced a similar motion in a horizontal hammock-wise suspension of the subject [3]. The scientific research into BCG surged later in the mid-1900s, an era known as the golden age of BCG [4,5]. The most prominent pioneer in this regard was Isaac Starr, who published what is considered the first scientific works in this field [6] and later headed the Committee on Ballistocardiographic Terminology [7]; since his first publication, he continued to investigate BCG for decades [5]. Starr was the first to present a model of the signal’s commonest morphology with a proposed terminology [6], which was later adopted and promoted by the aforementioned committee [8], Figure 1 depicts the signal with the terminology proposed by Starr at that time, which was later elaborated on by other researchers, notably by reporting preceding F and G waves [9,10]. However, the most prominent segments of the signal are H–L, which represent the systole phase of the cardiac cycle and should be distinguishable in a healthy subject under normal circumstances, since the non-systolic components are less perceptible, especially with a fast heart rate, when superimposition of waves is evident [4].

The body of research at that time aimed at exploiting the technique’s potential for medical diagnosis; however, the lack of consensus among researchers about the physiological genesis of the signal’s morphology, the failure to adhere to standardized techniques for measurements, and the ambiguity in interpretation of the results, given the inherent great level of scrutiny a medical diagnostic tool is naturally placed under, led to its effective dismissal from the medical community in the late 1970s [11]. In the last two decades, however, the research community has revived its interest in BCG with substantial momentum. Several factors have contributed to this phenomenon. Most notably, the BCG has a great capacity to be realized in an unobtrusive sensory form and be embedded with different configurations in the patient’s environment. Secondly, owing to its physical nature, it is able to convey key medical information about the cardiovascular system, which might be otherwise unattainable [10], e.g., the force of the heart’s contraction, which is a key indicator of the heart’s physiologic age and its decline could be an early warning of a heart disease [12]. Furthermore, the recent technological advancements in sensory fabrication, signal processing, and computational powers facilitated the resurgence of BCG.

Considering the nature of BCG being the measurement of the body motion that comes about to oppose the motion of mass within it, primarily blood, as Newton’s third law of motion dictates, and the fact that the motion of blood in the body is not confined in a single direction, making BCG practically omnidirectional, this made it possible to measure BCG along several directions. Consequently, the Committee on Ballistocardiographic Terminology recommended three standard spatial axes of measurement: longitudinal (head-foot), transverse (side to side), and dorsoventral or anteroposterior (back to chest) [8]. However, it has been reported that a BCG measured in the longitudinal direction, particularly of a bedridden subject, has stronger cardiac output components than those measured in the other directions [13,14]. Additionally, the signal’s morphology of a transverse or a dorsoventral BCG is highly dependent on the subject and the subject’s position on the bed, more so than that of a longitudinal BCG, and, by the same token, the morphological variability of longitudinal BCG is lesser than that of the others [14]. Hence, unlike most modern bed-embedded BCG systems, the BCG in this work is set up to be measured along the longitudinal direction in an attempt to obtain the best possible signal to work with.

Since there are different ways to take account of the reaction motion of the body; namely, via displacement, velocity, or acceleration, and with each of these measurands being measurable through various sensor modalities, ballistocardiographers have experimented with various BCG systems throughout the decades. Furthermore, in earlier times, notwithstanding all setups being table-based, they differed with regard to the implemented technology and the resulting setup’s natural frequency, but they were usually labelled according to the latter. To name a few, the earliest BCGs, by Gordon [3] and Henderson [15], were deemed ultra-low frequency BCGs, on the other hand, Starr’s spring-coupled undamped table produced high-frequency BCG [6], and Nickerson’s apparatus, capable of continuously adjustable damping, presented low-frequency BCG under critical damping [16]. Later, Dock introduced a different measurement process that provided direct body BCG, however, he kept the table-based setup [17]. Each of these types produced a different common BCG waveform [8] while attempting to address drawbacks or forms of distortion that other types may have suffered [4].

As mentioned previously, when the potential of the BCG started to be recognized in the medical field, it was subjected to great scrutiny to substantiate its value as a cardiological examination. This necessitated the aforementioned classification of BCG systems, as they appeared, and the standardization of their respective morphologies because medical diagnosis needs above all to establish a norm to contrast anomalies against. Nevertheless, with the recent interest in BCG shifting its objective towards ubiquitous unobtrusive monitoring, the recent novelties in the field manifested mainly in the implemented sensors, the manner of embedding sensors in patients’ surroundings, and the techniques and algorithms employed in signal processing and data extraction. In this respect, BCG measurements have been installed in weighing scales [18,19,20], which have the benefit of pure longitudinal BCG, however, practical constraints on long measurement times is a major disadvantage. Additionally, chair-based BCG is popular among researchers, where BCG transducers can be embedded in the seat [21], backrest [22], or even headrest [23]; moreover, equipping wheelchairs with cardiac monitoring has also been explored [24]. At the same time, the widespread utilization of BCG in sleep monitoring applications, made embedding BCG sensors in beds quite prevalent [14,25,26,27,28], even in air mattresses [29] and pillows [30].

The BCG transducers found in recent literature are even more diverse. For example, we find film sensors, like electromechanical film (EMFi) [22,24] and polyvinylidenefluorid film (PVDF) [21,25]; strain gauges, which are dominant in but not limited to weighing scales [14,18,19,20]; MEMS accelerometers [24]; fiber optical sensors [23]; piezoelectric pressure sensors [27]; hydraulic pressure sensors [26]; and pneumatic systems based on air pressure sensors employed in elaborated configurations [28,29]. It is worth pointing out that this variety of transducers has further increased the variability of the BCG waveform, which, for the most part, still relates to the conventional waveform depicted in Figure 1, however, significant alterations can often be noticed.

Furthermore, given the morphological nature of the BCG signal distinguishing individual heartbeats in it is not usually straightforward, unlike, for example, detecting the sharp QRS complex in ECG. Thus, various methodologies are constantly being developed, some are as simple as an adaptive threshold to detect the IJ segment (Figure 1) [19], or a basic peak detection algorithm applied after back-and-forth transformation between time and frequency domains for signal screening and cleaning [23]. On the other hand, BCG measurement setups with their resulting waveforms usually required more sophisticated approaches, which have made use of, among others, unsupervised machine learning techniques like hierarchical clustering [27], or k-means clustering [26], which in Reference [14] was part of an elaborate detection algorithm based on three different methods, preceded by a training phase: cross-correlation, modified k-means clustering, and heart valve function, which was concluded by weighting each method’s outcome through reliability functions. Other useful tools often found in the literature are the continuous wavelet transform (CWT) [20] as well as the discrete wavelet transform (DWT) [31].

In this paper, the longitudinal BCG that is measured through a highly reliable, and readily available sensor, in the form of a load cell installed on a bed, is assessed, and the likelihood of successful heart and respiration rates monitoring through it is evaluated. An unsupervised machine learning method is employed for heartbeat monitoring, while its performance is referenced to an ECG signal that serves as ground truth. The algorithm is a modified version of the one found in Reference [27], which reported a successful detection rate of 49.2% of recorded heartbeats. The main purpose of this project is to improve the heartbeat recognition rate and to investigate the influence of lying positions on recognition probability.

## 2. Materials and Methods

### 2.1. Measurement Setup

The measurement system is comprised of a force sensor to measure body motions, namely, those induced by cardiac and respiration activities; an ECG sensor, which establishes a reference for the evaluation of the heart rate detection; and a data acquisition tool.

#### 2.1.1. Force Sensor

The force sensor utilized to acquire the BCG signal is convenient in a number of respects. It is a low-cost, off-the-shelf load cell that is usually utilized in weighing scales (3D-Mechatronics, Bremen, Germany). It is practically very reliable with low demands on maintenance. It comprises an aluminum beam with a hollowed section in the middle, making it less resistant to bending forces, and two strain gauges placed on either side of the hollowed part, forming a two-element bridge circuit. This Wheatstone bridge setup has a very reasonable linear response and sensitivity. The load cell is interfaced through an electronic circuit based on the 24-bit HX711 analog-to-digital converter (AVIA Semiconductor, Xiamen, China). The transducer is suited for up to around 50 N (5 kg) of load, which when calibrated was found to have a theoretical resolution of about 24 µN per A/D count over its operational range. The accompanying circuit with the built-in oscillator is capable of providing up to an 80 Hz sampling frequency.

**Figure 2:**
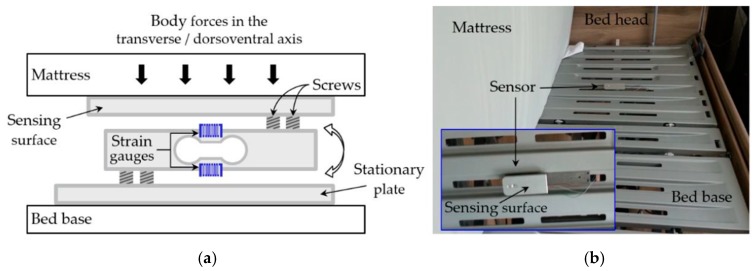
The Load cell setup to measure the body forces in the transverse/dorsoventral axis for comparison with the signal in the longitudinal axis: (**a**) The load cell structure diagram; (**b**) the actual sensor placement relative to the bed; the mattress is lifted temporarily for the sake of the picture.

**Figure 3:**
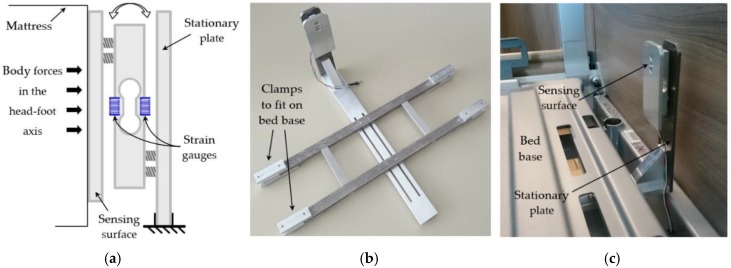
The load cell setup for the longitudinal BCG: (**a**) The load cell structure diagram; (**b**) the complete mechanical structure; (**c**) the structure fitted at the top side of the bed base. The clamps fit on the bed base from underneath.

The influence of the force sensor placement on the resulting signals was initially investigated. For this reason, a mechanical structure was designed, which sandwiches the load cell between two parallel plates, with the load cell beam being fixed to each plate at its opposing sides. Firstly, in order to obtain the transverse/dorsoventral signal the sensor was placed horizontally between the mattress and the bed base, below the upper part of a volunteer’s body, similar to the placement of most modern bed-embedded BCG sensors. The upper plate, facing the mattress, is designated as the sensing surface, while the lower one, resting on the bed base, as the stationary plate. Figure 2 (a) depicts the sensor’s structure diagram in this configuration, while (b) shows the actual placement of the sensor in relation to the bed. Secondly, in order to measure the BCG signal in the longitudinal direction, the sensor was set up vertically to sense the body motions transmitted through the mattress at its top side. In this configuration, which is the one adopted for this monitoring system, the stationary plate is made as such by a structure that is attached firmly to the slotted bed base, while the sensing surface faces the top side of the mattress. Figure 3 (a) displays the structure diagram of this configuration, (b) the complete mechanical structure for the longitudinal measurement, and (c) shows the structure fitted on the bed.

#### 2.1.2. ECG Sensor

The ECG signal is recorded by means of a single-lead heart rate monitor board (SparkFun Electronics, Niwot, CO, USA), which has an integrated signal conditioning chip intended for ECG measurement, named AD8232 (Analog Devices, Norwood, MA, USA). This tool provides the ECG signal in 0–3.3 V analog form; therefore, its conversion to digital form is carried out by the data acquisition system.

#### 2.1.3. Data Acquisition

**Figure 4:**
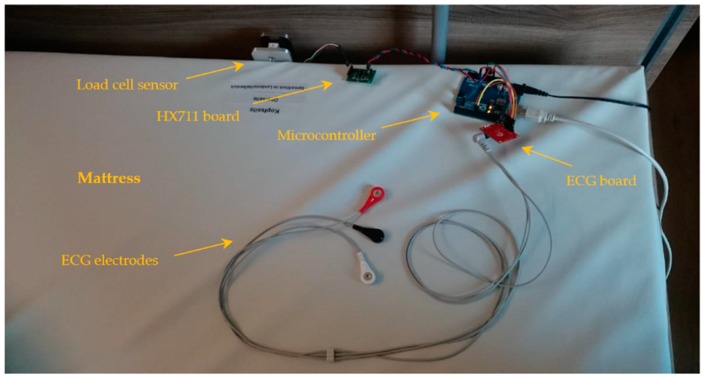
The complete measurement system.

The two sensors are interfaced through an Arduino Uno microcontroller (Arduino AG, Somerville, MA, USA); Figure 4 shows the complete measurement system. The microcontroller reads the force sensor by communicating with the HX711 chip through the Serial Peripheral Interface (SPI) at the maximum available sampling frequency, dictated, as mentioned, by the HX711 chip. The sampling frequency in the case of the board at hand is around 84.8 Hz. On the other hand, the analog ECG signal is sampled at three times that rate, i.e., 254.4 Hz. The microcontroller carries out the sampling at fixed time intervals with proper independent timestamping of both signals. Additionally, the force sensor is scaled to a force unit of millinewton (mN), as per previously established calibration parameters, before forwarding both measurands serially to the PC along with their timestamps. On the PC side, a desktop application organizes and saves the transmitted data, namely, the Processing Integrated Development Environment (IDE) (Processing Foundation, processingfoundation.org).

### 2.2. Signal Preprocessing

**Figure 5:**
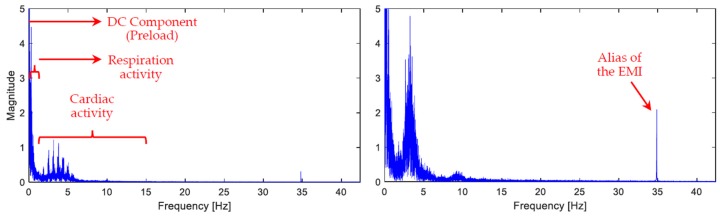
Frequency spectrum diagrams of raw BCG signals taken from two volunteers, both lying in the supine position. The major signal components are annotated. The upper parts of the diagrams have been truncated since the magnitude of the DC component (the preload) extends to the thousands. The clear difference in the distribution of the frequency components of the two signals reflects the variability of the BCG waveforms across people. The significant variation in amplitudes overall between the two signals will be discussed later. Given that the Nyquist frequency (half the sampling frequency) is 42.4 Hz, an alias of the EMI (50 Hz) appears at 34.8 Hz.

The force sensor in its basic setup delivers a signal containing various components. Naturally, it senses all motions induced by the body, whether caused by cardiac or respiratory activities, or any voluntary muscle movements. Additionally, depending on the surroundings, vibrations through the floor might be picked up in the signal, not to mention the electromagnetic interference (EMI), and the DC component present due to the necessary preloading by initial contact with the mattress. Figure 5 displays two frequency spectrum diagrams of the sensor’s raw signal that are truncated for the convenience of display. These two sample signals were taken from two volunteers lying in the supine position; both are depicted to showcase the variability of the BCG waveform even in the frequency domain. In order to separate the sought-after components from the aforementioned artifacts as well as from one another, signal filtration is implemented digitally by means of Matlab (The MathWorks, Inc., Natick, MA, USA).

**Figure 6:**
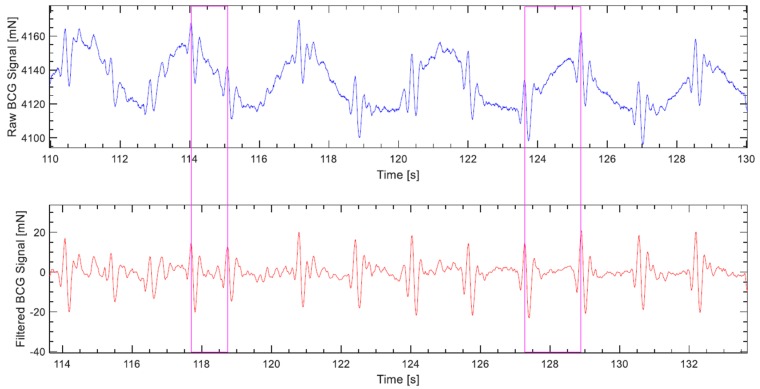
A sample load cell signal. The upper plot shows the raw signal, while the lower one shows its filtered counterpart. The filtration is carried out by high- and low-pass *fircls1* filters with 0.5 and 20 Hz cutoff frequencies, respectively. The magenta lines’ intersections with the time axes highlight the delay caused by filtration; about 3.7 s.

The reported frequency bandwidth of interest for the cardiac component varies among publications, where the cutoff frequencies range between 0.1–1 Hz for high-pass filters, and 10–25 Hz for low-pass filters [32]. Previously, namely in Reference [33], we assumed the bandwidth of 0.5–20 Hz and carried out the filtration accordingly; however, since then, with further experimentation and new measurements taking place, the bandwidth 0.5–15 Hz, was found empirically to yield overall more fruitful results as far as heart rate monitoring is concerned. Moreover, the BCG signal is obtained by implementing finite impulse response filters (FIR), which are advantageous in the sense that they do not induce any phase distortion in the filtered signal because they maintain a perfectly linear phase response. Specifically, the *fircls1* filter function is used, which is an inbuilt function in Matlab that facilitates additionally the fine-tuning of the levels of ripple and attenuation in the passband and stopband, respectively. The lengths of the low- and high-pass filters were 80 and 540, respectively; whereas the levels of ripple and attenuation in both filters were set to 0.005% and 0.01%, respectively. In the upper plot of Figure 6 a sample raw load cell signal is plotted, while its realigned filtered counterpart is shown in the bottom plot.

On the other hand, the respiration component is obtained through a single 3rd order band-pass Butterworth filter that belongs to the infinite impulse response filters (IIR) category, which is also an inbuilt function in the Matlab environment called *butter*. This filter is designed to have a passband that is as flat as possible, meaning minimum ripple, while retaining decent phase linearity throughout that passband. Additionally, as an IIR filter, it requires less computational power and induces less delay in the signal. The adopted bandwidth for this component also varied in BCG-related publications; however, more medically specialized research has reported that the normal respiration rate of elderly people above the age of 65 is 8–30 respirations per minute (2.5–97.5 percentile), while younger people have a narrower distribution, however well within these boundaries [34]. Those rates correspond to a frequency bandwidth of 0.13–0.5 Hz; nevertheless, the employed band-pass filter has a slightly wider passband with the cutoff frequencies 0.05 Hz and 0.5 Hz.

Even though the ECG signal is more susceptible to noise, especially when a volunteer lays prone over the electrodes, and considerable levels of noise are sometimes encountered, yet the QRS complex remained distinct enough during recorded measurements, hence, the signal has not been filtered by any means.

### 2.3. Heart and Respiration Rates Detection Algorithms

All the detection algorithms and the evaluation of their outcome reported here are implemented through Matlab.

#### 2.3.1. Heart Rate in BCG

Keeping in mind the variability of the BCG signal’s morphology across people, lying positions, and even with time, a versatile algorithm that is independent of history, at least apart from the immediate one, and capable of perceiving the short-term slightly changing pattern of the BCG cardiac cycle was required. Consequently, the choice of utilizing an unsupervised pattern recognition algorithm that operates immediately without a training phase was made. Hence, an improvement over the agglomerative clustering algorithm put forward by Paalasmaa in [27] is attempted. In Appendix A further explanations for some of the terms related to this algorithm can be found.

**Figure 7:**
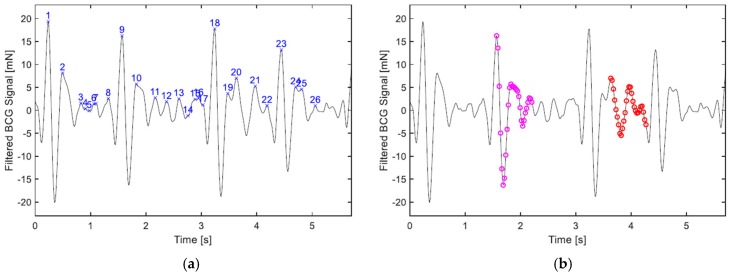
A segment of a BCG signal being processed by the clustering algorithm, which is shorter than the usual case for convenience of display: (**a**) local maxima are identified; (**b**) two of the twenty-six feature vectors contained in the segment are highlighted, namely, vectors 9 and 20, with each one comprising 28 features, i.e., signal samples.

The BCG signal is run through the algorithm in segments, each with a length of 10.66 s; effectively it is 10 s since there is 0.66 s of overlap between successive operations. Firstly, the derivative of the signal is calculated, through which the local maxima that presumably represent the upward waves in the signal (H, J, L, etc., see Figure 1) are identified, this step is depicted in Figure 7a. Afterwards, feature vectors are formulated. The feature selection here is straightforward; each feature vector begins with a local maximum point and contains the segment of the signal that follows.

The temporal length of the feature vector, i.e., the time the signal segment contained in the vector covers, is 0.66 s, which corresponds to the time interval of a 90-bpm heart cycle. Reducing this parameter risks losing important information, making the wave represented in the vector less distinctive from other types of waves; however, increasing it might unnecessarily increase algorithm computations. Furthermore, to keep a minimum information redundancy within the feature vector, a down-sampling by a factor of 2 is employed, meaning, the feature vector contains every other sample of the signal segment. Figure 7b highlights two of the feature vectors being processed, as examples. The downsampling reduces the computational time without compromising the overall process. Since the sampling frequency at hand is high enough to contain frequency components up to 42.4 Hz, as per the sampling theorem, and given that the BCG signal is low-pass filtered at 15 Hz, a downsampling to half the original sampling frequency will still preserve all the frequency components of interest in the signal. As a result, each feature vector is comprised of 28 features to cover the aforementioned temporal length.

The proximity measure, according to which the feature vectors are clustered, has to handle the varying amplitudes of the BCG waves across different heartbeats with minimal differentiation. Therefore, the inverse cosine dissimilarity measure is quite apt from this perspective, since it is a pure angular measure. Nevertheless, some boundary conditions are compounded with the dissimilarity measure, which, on the one hand, avoid computations deemed practically unnecessary, and on the other hand, can be very vital for proper clustering under certain circumstances. If any of the conditions is fulfilled, the angular dissimilarity between the pair of vectors in question is given the maximum value of π (rad). Those conditions are:

1. If the ratio of the feature vectors’ magnitudes to one another is larger than *α*, or, as the case may be, smaller than *1*/*α*, where *α* is set to 3.
2. If the difference between the timestamps of the respective vectors’ first elements is less than *t_min_*, which is set to 0.33 s, making the algorithm only suitable for heart rates lower than 180 bpm.
3. If the absolute difference in the chronological order of the vectors is less than *i* (index).

The third condition relies on the fact that, as mentioned earlier, the BCG waves H–L dominate the signal and should usually be distinctively observed forming a W shape [4]. Consequently, it should be reasonable to assume that a single cardiac cycle must contain a minimum of three upward waves, meaning *i* = 3, which is valid most often; however, when the heart rate is high enough, sometimes superimposition of waves is strong, and the BCG waveform does not assume more than two local maxima. When that is the case, having this condition with *i* = 3 is detrimental to the clustering because it will prevent waves of the same type from being clustered together. One solution is to perform the whole clustering procedure twice, first with *i* = 3, then *i* = 2, afterwards the consistency of the detected beat-to-beat heart rate is assessed for both runs via a standard deviation function, and if *σ* when *i* = 2 is less, then the result of that clustering is adopted, otherwise the result of *i* = 3 remains.

After combining the inverse cosine dissimilarity measure with the aforementioned boundary conditions, the overall dissimilarity function becomes:

(1) $$d\left(x,\;y\right)=\left\{ \begin{array}{cc} \cos^{-1}\left(\frac{x^{T}\;y}{\left\|x\right\|\;\left\|y\right\|}\right), & \mathrm{if}\;\frac{1}{\alpha }\leq \frac{\left\|x\right\|\;}{\left\|y\right\|}\leq \alpha \;\mathrm{and}\;\left|t_{x_{1}}-t_{y_{1}}\right|\geq t_{\min}\;\mathrm{and}\;\left|i_{x}-i_{y}\right|\geq i\\ \pi , & \mathrm{otherwise} \end{array}\right.$$

where x and y are the pair of feature vectors, the dissimilarity between which is being quantified.

**Figure 8:**
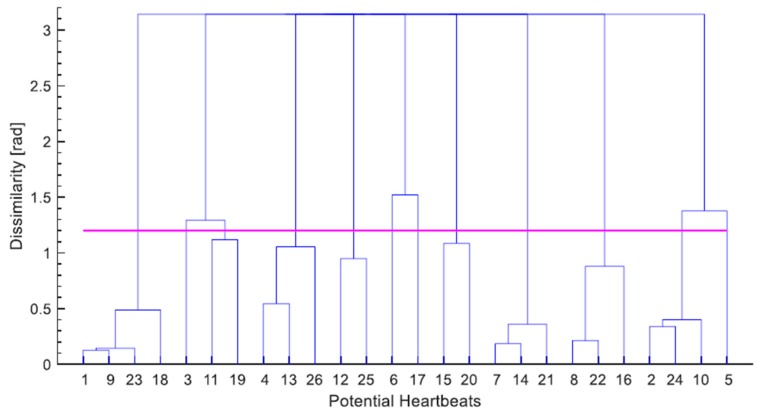
A dendrogram showing the complete hierarchical clustering of the sample signal presented in the previous figure. The magenta line represents the dissimilarity threshold (θ = 1.2) that is used to determine the final clustering.

The complete-link hierarchical clustering scheme begins with each vector forming a single-element cluster, then merges these clusters together, two at a time, until all vectors are contained in a single cluster. Figure 8 depicts a dendrogram that displays the complete clustering hierarchy of the sample signal shown in Figure 7. When the dissimilarity between a pair of clusters, at least one of which contains more than a single element, is evaluated, the complete-link algorithm adopts the max proximity function [35] (p. 620), that is:

(2) $$d_{\max}\left(C_{i},\;C_{j}\right)=\underset{x\in C_{i},\;y\in C_{j}}{\max}d\left(x,\;y\right)$$

where C_i_ and C_j_ are the pair of clusters in question. Therefore, the dissimilarity between clusters is fully determined by their respective most dissimilar vectors. This is crucial for the conditional dissimilarity function implemented here, otherwise a min proximity function, for instance, would render those boundary conditions practically void.

To conclude the hierarchical clustering meaningfully a threshold for the angular dissimilarity, θ, is employed. The clustering that occurs up to this level of dissimilarity determines the final clusters, and beyond which any clustering is discarded. This threshold was empirically tuned to a value of 1.2 (rad); the magenta line in Figure 8 depicts this parameter. The final step of the algorithm is choosing a cluster that reflects the cardiac cycles best. Two criteria are utilized for this purpose, the second of which comes into effect, only if the first was indecisive:

1. The size of the cluster. The cluster containing the largest number of vectors is chosen.
2. The mean of dissimilarity. If the largest number of vectors contained in a cluster is equally matched by more than one cluster, the one with the lowest mean of dissimilarity among its vectors is picked.

In the dendrogram of Figure 8, considering the clusters formed below the dissimilarity threshold, it can be clearly observed that the largest cluster, which is the only one containing four vectors, is the first one to the left; therefore waves 1, 9, 18 and 23, which happen to be all the J waves present in the segment are chosen to represent the cardiac cycles.

#### 2.3.2. Respiration Rate in BCG

The scope of this paper with regard to respiration is primarily to determine whether the respiration component in the load cell signal is present with sufficient magnitude that facilitates monitoring it.

**Figure 9:**
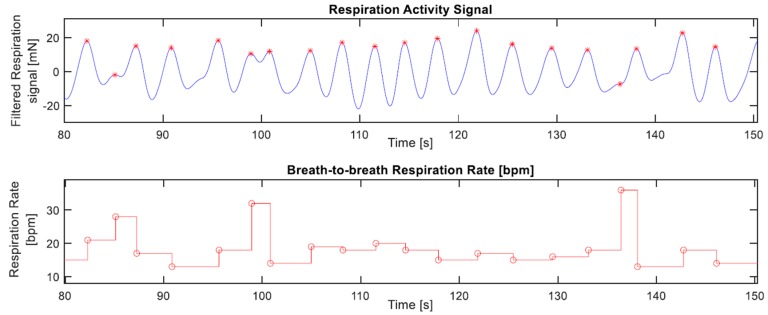
Sample of the workings of the respiration rate calculation method. The upper plot depicts a respiration signal with red asterisks marking the detected local maxima, i.e., the inhalation peaks, while the corresponding calculated breath-to-breath respiration rate is shown in the lower plot.

Given that the narrow bandwidth of the respiration component lies at the lower end of frequencies, where the motions of respiration dominate, unchallenged by the usual noise sources, this simplified the algorithm significantly. Consequently, using its derivative, the local maxima of the respiration signal are identified in order to mark the boundaries of respiration cycles and calculate the respiration rate accordingly, Figure 9 depicts a sample output of this procedure.

#### 2.3.3. Heart Rate in ECG

**Figure 10:**
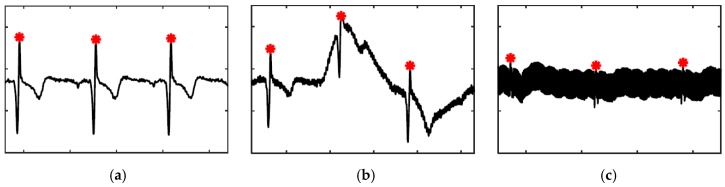
ECG signal samples with QRS complexes marked with red asterisks: (**a**) the common form; (**b**) baseline fluctuation; (**c**) large EMI.

For the most part, a simple peak detection with an absolute threshold works well to detect the QRS complex in ECG signals; Figure 10a depicts the common form of the signal. However, two types of noises have been encountered, usually due to poor electrode connection, which require more elaborate algorithms: the first one is a deviation of the baseline of the signal, shown in Figure 10b, which is addressed by calculating the immediate relative increase in amplitude at each peak and setting a threshold for that measure; and the second one is a considerable amount of EMI, shown in Figure 10c, which is rarely encountered in the depicted intensity; it is handled by comparing the relative increase in amplitude at a peak with a moving average of the same measure of other peaks surrounding the peak in question, fine-tuned by a coefficient that is multiplied by the moving average.

### 2.4. Measurements

The measurements involved seven volunteers, who have approved of and signed a consent form permitting the publishing of the results based on their measurements.

Each of the volunteers lay in the four commonest lying positions: the supine, prone, left lateral, and right lateral. In the supine and prone positions, the volunteers lay flat on the mattress without a pillow, whereas a pillow was provided when they lay on their sides. During the measurements the volunteers were asked to lie still; however, small movements taking place every now and then were inevitable. In each measurement, BCG and ECG signals were recorded continuously for five minutes in a certain position, then repeated twice in the same position. This division was meant to allow a short pause for the volunteer to move between the measurements if they wished to do so. Therefore, the measurements added up to one hour of measurement time for each volunteer, and seven hours in total.

At the beginning of the measurements, a good coupling of the sensing surface with the mattress after a volunteer lay down on it was ensured by checking the amount of preload the load cell was reading. It is worth mentioning that the measurements presented here were carried out in two stages, which involved two and five volunteers, respectively. In the first stage, the preload was kept between 4–5 N, whereas in the second one, it was between 12–21.5 N. This variation had an influence on the magnitudes of the transmitted forces, which will be discussed later. Of course, the fact that the operating range of the load cell is up to 50 N provides a considerable margin for maneuvering.

### 2.5. Evaluation

**Figure 11:**
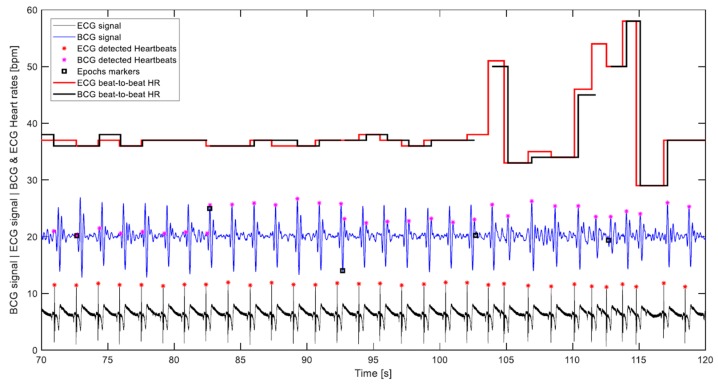
Visual evaluation diagram that contains both BCG and ECG signals, which have been rescaled and shifted for the convenience of display; their respective detected heartbeats and corresponding beat-to-beat heart rates; and the signal epochs’ markers that highlight the segment’s divisions, by which the signal was handled. This volunteer has been diagnosed with heart arrhythmia.

The effectiveness of the heart rate detection algorithm was evaluated based on the seven hours of measurements time. In this respect, few things are worth pointing out:

- The correct detection was evaluated visually, which was carried out by plotting the BCG and ECG signals with their respective heart cycles’ detection markings and corresponding calculated heart rates. Figure 11 displays part of a signal undergoing visual assessment.
- The calculated heart rates were rounded to integers. Furthermore, a discrepancy of up to 5 bpm between the ECG and corresponding BCG beat-to-beat heart rates was considered a correct detection, as long as it was visually determined that the detected BCG waves of the consecutive heart cycles are of the same type, which meant that the discrepancy was not a result of wrong detection, but rather a time shift of the wave due to an artifact, for instance. Such high discrepancies happen infrequently, and mostly with higher heart rates.
- Given that the algorithm does not single out any specific wave to represent the cardiac cycle, like the J wave for instance, but rather looks for the waveform most consistently occurring in the 10-s segment being processed. It follows that the calculation of the beat-to-beat heart rate of the cardiac cycle that lies between two consecutive segments based on potentially different waves is not warranted. In Figure 11, which depicts 5 transitions between 10-s segments, it can be noticed that the algorithm was first detecting the H wave, then in the next segment the J wave, and later the L wave. Consequently, the 29 intermediate cardiac cycles, which lie between the 30 segments of a 5-min measurement are subtracted from the number of cycles assumed to be detectable by the algorithm, and the correct detection percentage is calculated accordingly. This can be observed in the plot of Figure 11 by the cuts in the BCG heart rate step diagram. It is worth pointing out that this exclusion was not entertained in the previous paper, namely Reference [33], and all heart cycles contained in a measurement were considered detectable.
- Despite the fact that artifacts caused by volunteers’ movements occurred, since it was not that frequent, no exclusion of heartbeats that might have been undetected because of such artifacts was made in this evaluation.

## 3. Results and Discussion

**Figure 12:**
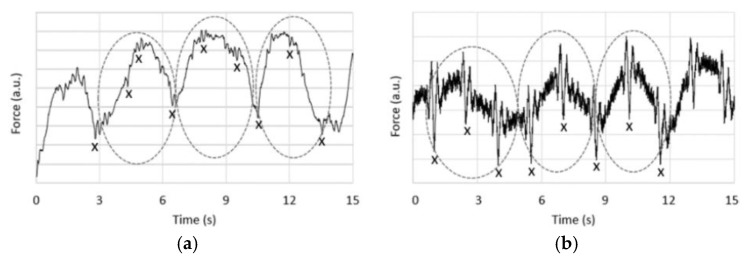
Two samples of raw load cell signals measured for a volunteer in a supine position with the sensor positioned (**a**) below the mattress (see Figure 2); (**b**) at the top side of the mattress (see Figure 3). Respiration and cardiac cycles are marked by circles and “x”, respectively.

The preliminary measurements that were carried out to assess the influence of the force sensor placement on the signals revealed that the respiration component generally dominates the transverse/dorsoventral signal, i.e., when the sensor is placed under the mattress (see Figure 2). This can be seen in Figure 12a by the magnitudes of the respiration waves marked with circles when compared to the much smaller BCG waves marked with “x”. Whereas in Figure 12b, which corresponds to the longitudinal BCG setup (see Figure 3), the magnitudes of the respiration and BCG components are much more comparable. This improves the quality of the BCG signal by facilitating a more optimized signal amplification and more effective filtration of the respiration component.

The results of the evaluation of the heart rate detection algorithm are presented in Table 1, which relates some of the volunteers’ biometrics and the measurements’ details to the rates of correct detection. The measurements of the different lying positions for each volunteer are grouped together; the rate of detection for each position is given as well as the average rate for all positions of each volunteer. The overall correct detection rate was 83.9%, which is the average of the detection rates of all measurements, given that they all have the same weight, that is, their equal length of time.

The first two volunteers in the table had excellent detection rates in all positions, whereas the other five usually had at least one position with a relatively worse rate of detection. This shows that there is an influence of the lying position on the BCG signal quality. For an overview of how the algorithm performed with the four lying positions Table 2 presents the average rate of detection for each lying position across all volunteers. It can be said that the left lateral position had a distinctly higher detection rate than the other positions, which fared rather the same.

Moreover, the second volunteer, who is diagnosed with heart arrhythmia, had some of the highest detection rates, which shows that the algorithm handles the sudden change in the time interval of a heartbeat very well. A sample of his arrhythmic pattern is depicted in Figure 11. On the other hand, the algorithm was not as successful when dealing with the signal of the last male volunteer in the table and produced overall the worst detection results. This was anticipated, given his high body mass index that seems to have a detrimental effect on the BCG signal. Interestingly, the last volunteer in the table, who is an elderly female with four bypasses in her heart, gave reasonably good results, except in the supine position, which fared the worst among all measurements, with one-third of the heartbeats being correctly detected. Perhaps the bypasses interfered most with the signal in that particular position.

**Figure 13:**
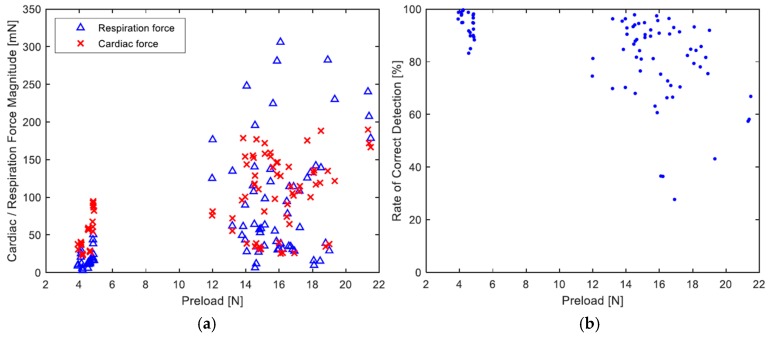
(**a**) The relation between the sensor preload and the magnitude of the corresponding cardiac and respiration forces. The respiration force magnitude is evaluated by its average peak-to-peak amplitude, whereas the cardiac force is assessed based on the standard deviation of the signal sample points since the signal generally assumes a Gaussian distribution; its magnitude is determined as 6 σ. (**b**) The relation between the preload and the resulting rate of correct detection.

The longitudinal sensor setup presented here uses the mattress as a medium for the transmission of body forces in a manner that introduces the preload on the sensor as an independent parameter, which evidently has some influence on the transmitted signals. As mentioned in the measurements section, a correlation between the magnitudes of the preload and the measured force, and by extension, its cardiac and respiratory components, was revealed; Figure 13a depicts this correlation. In the figure, if grouped according to the amount of preload, two groups of measurements can be observed. The small group to the left, which had much smaller preloads, resulted in the whole in smaller magnitudes of forces. However, in the group to the right, notwithstanding the greater dispersion, the higher preloads resulted in overall higher magnitudes.

It is worth pointing out that the proportionality of the cardiac and respiration magnitudes to one another, in the same measurement, differed across volunteers, as well as lying positions of the same volunteer. From Figure 13a, it can be discerned that with smaller preloads the cardiac component was generally stronger, which was not usually the case with higher preloads, especially since the respiration forces, marked with blue triangles, showed greater dispersion than the cardiac forces. As a consequence of this observation it might be said that the more the mattress is pressed against the sensing surface, the better the coupling between the two is. On the other hand, however, when the relation between the preload and the rate of detection is considered, which is portrayed in Figure 13b, it seems that lower preloads gave, as a whole, higher detection rates. Nevertheless, since the lower preloads involved only two volunteers, such a conclusion might be viewed statistically as premature.

The types of false detections that occur in this clustering algorithm can generally be classified into three categories: false positive, which is the detection of more than a single wave within a single cardiac cycle, i.e., multiplicate detection; false negative, when not a single wave is detected within a cardiac cycle; and misplaced detection, which is the detection of a single wave in a cardiac cycle that is different from the majority of detected waves in the other cardiac cycles within the segment being processed. It is worth pointing out, that the dissimilarity threshold, θ, does not have a direct influence on the third class of false detections the way it has on the other two classes. As may be discerned from the dendrogram shown in Figure 8 the value of θ determines the size of the final clusters, and by extension, the cluster chosen to represent the heartbeats. Therefore, increasing θ may lessen the false negative detection on the one hand, but it may increase the false positive one on the other hand.

In the work we presented in Reference [33], the third boundary condition of the dissimilarity function had not yet been implemented. It may be argued that there is an overlap of the effects the second and third conditions have in the function, which is generally true; nevertheless, the need for an improved algorithm became obvious, when it made a large amount of false positive detections in a BCG signal of a volunteer lying in a certain position, even when lower values of θ where attempted.

**Figure 14:**
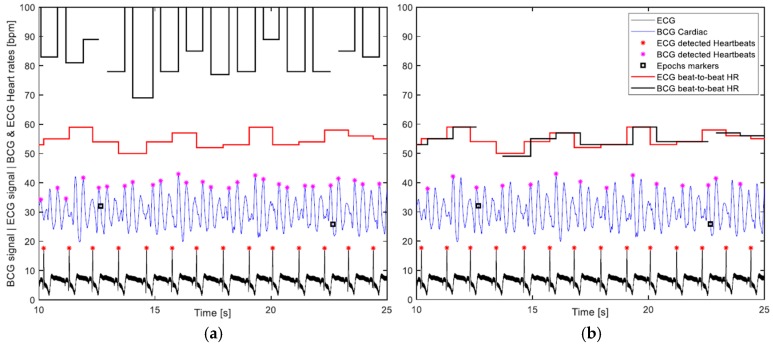
The performance of the detection algorithm with a peculiar sample signal (**a**) when processed without the lately added boundary condition resulted in excessive positive detections, whereas in (**b**) the issue is resolved solely by this addition; all other parameters are kept the same.

The benefit of adding the third condition was two-fold. Firstly, it suppressed most of the false positive detections happening at the previously employed θ (in Reference [33] it was 1 rad) across all measurements. Secondly, as a result, it facilitated raising θ (to 1.2 rad), which decreased the probability of false negative detection as well, improving the overall detection rates. This can be recognized to a certain extent by comparing the results of the two volunteers, whose same measurements were handled by the two versions of the algorithm; the first two volunteers in Table 1 of both publications, respectively. Furthermore, the effect of adding this new boundary condition can be directly observed in Figure 14, which depicts the algorithm’s detections with a same sample signal before and after this addition, all other parameters, including θ, are kept the same. In plot (a) almost all heart cycles shown had duplicate detections, while in (b) each cycle is detected only once.

Finally, it is worth pointing out that, as mentioned in the algorithms section, at higher heart rates superimposition of waves takes place and the BCG waveform might occasionally include only two upward waves in a cardiac cycle. Consequently, if the minimum index difference was set to three, the presence of even a single such cardiac cycle within the segment being processed would cause incorrect clustering for the majority of waves in the segment, hence the solution of carrying out the clustering with two values of the minimum index difference was implemented. However, this solution requires further improvement, and to minimize the computations of the algorithm in the future, a better solution would be to devise a proper measure for assessing the outcome of the first clustering, then carrying it out a second time only if the first outcome is not satisfactory.

## 4. Conclusions

In this work, the implementation of a low-cost force sensor as a BCG and respiration sensory element is investigated. A preliminary setup and integration scheme, measuring the longitudinal BCG of patients on a bed, is proposed and its raw signal is compared to the BCG setup (transverse/dorsoventral) most commonly found in today’s bed-embedded BCG systems. To evaluate the usefulness of the proposed setup for heart rate monitoring, the ability to correctly recognize the individual heartbeats contained in the filtered BCG signal is tested by an unsupervised machine learning algorithm and referenced to the ECG. The algorithm, which was originally proposed by another research group [27], is further improved, thoroughly discussed, and tested with seven volunteers and four lying positions. The overall detection rate achieved was 83.9%.

Since the setup depends on a mattress as a medium for the transmission of forces, the preload aspect of the initial sensor-mattress contact is briefly discussed and some preliminary correlations are presented; admittedly, however, more tests and statistical analyses are recommended for the future in order to thoroughly understand this interaction. By the same token, as suggested, there is always room for further refinement of the algorithm itself.

## Figures and Tables

**Table 1:** BCG measurements’ details and their corresponding heart rate detection evaluation.

Volunteer Details				Lying Position	Average Heart Rate [bpm]	No. Theoretically Detectable Beat-to-Beat Cycles	No. Cycles Not Correctly Detected ^3^	No. False Positive Detections ^4^	Correct Detection (%)	Avg. Corr. Detection (%)
Sex	Age [yr]	Wt. [kg]	Ht. [cm]							
M	31	83	180	Supine	76	1050	19	0	98.2	95.4
				Prone	64	872	100	0	88.6	
				Left side	69	944	32	0	96.6	
				Right side	68	928	18	0	98.0	
M ^1^	35	78	180	Supine	39	502	29	8	94.3	93.0
				Prone	41	524	19	4	96.4	
				Left side	38	486	43	8	91.2	
				Right side	43	551	55	7	90.0	
M	28	75	170	Supine	53	700	65	1	90.7	85.3
				Prone	56	756	149	5	80.5	
				Left side	57	758	37	2	95.0	
				Right side	61	822	204	3	74.8	
M	30	78	170	Supine	87	1211	200	0	83.5	86.9
				Prone	83	1151	285	0	75.2	
				Left side	79	1089	67	1	93.9	
				Right side	82	1142	59	0	94.9	
M	26	65	170	Supine	59	802	75	1	90.7	86.4
				Prone	61	828	29	0	96.5	
				Left side	63	853	94	1	88.9	
				Right side	61	822	252	5	69.3	
M	30	95	173	Supine	84	1177	383	0	67.4	68.2
				Prone	88	1235	500	1	59.8	
				Left side	83	1150	173	0	84.9	
				Right side	86	1206	473	0	60.8	
F ^2^	70	67	158	Supine	68	926	615	9	33.6	72.2
				Prone	70	958	186	4	80.6	
				Left side	65	879	117	36	86.7	
				Right side	67	921	109	12	88.1	

^1^ The volunteer has been diagnosed with Heart arrhythmia; ^2^ The volunteer has four bypasses in her heart; ^3^ As a result of all three types of false detection mentioned in the text; ^4^ A false positive is the detection of more than a single wave within a single cardiac cycle.

**Table 2:** The average correct detection rates for lying positions across all volunteers.

Lying Position	Correct Detection (%)
Supine	79.8
Prone	82.5
Left side	91.0
Right side	82.3

## Appendix A

A clarification of some of the terms found in the machine learning algorithm section is provided in this appendix.

- Unsupervised pattern recognition:

Pattern recognition is the scientific discipline that aims to classify objects into a number of categories or classes. These objects, which are referred to by the generic term patterns, can be images or signal waveforms or any type of measurements that require classification. The unsupervised approach deals with problems, where the categories or classes, into which the objects are to be classified, are not known before the pattern recognition process takes place. Thus, the aim here would be to unravel the underlying similarities (or dissimilarities) among the objects and group (cluster) similar objects together, based on certain measures that quantify the similarities [35] (pp. 1–7).

- Agglomerative clustering:

A type of hierarchical clustering algorithm that begins with each object, i.e., its representative feature vector, forming a cluster on its own, and produces a sequence of clusterings of decreasing number of clusters at each step. That is, at each step a clustering is produced by merging two clusters from the previous set of clusters. The algorithm ends with all objects contained in one cluster [35] (p. 629).

- Features and feature vectors:

Features are a number of variables or measurable properties of an object (pattern). A feature vector is a convenient formation that contains these variables and should uniquely identify a single object; therefore, features must be properly selected so as to encode as much information about the object as possible. However, information redundancy among the features should be avoided. The representation of objects by feature vectors facilitates their classification based on quantifiable proximity measures [35] (pp. 5, 596).

- Proximity measure:

A proximity measure quantifies how “similar” or “dissimilar” two feature vectors are. Thus, a proximity measure is identified either as a similarity measure or a dissimilarity measure. The choice to implement one or the other depends on the application and the nature of the feature vectors [35] (p. 597).
